# Molecular dynamics trajectories for 630 coarse-grained drug-membrane permeations

**DOI:** 10.1038/s41597-020-0391-0

**Published:** 2020-02-13

**Authors:** Christian Hoffmann, Alessia Centi, Roberto Menichetti, Tristan Bereau

**Affiliations:** 1 Max Planck Institute for Polymer Research, 55128 Mainz, Germany; 2 Physics Department, University of Trento, 38123 Trento, Italy; 3 INFN-TIFPA, Trento Institute for Fundamental Physics and Applications, 38123 Trento, Italy

**Keywords:** Pharmacology, Chemical physics, Thermodynamics, Drug development

## Abstract

The permeation of small-molecule drugs across a phospholipid membrane bears much interest both in the pharmaceutical sciences and in physical chemistry. Connecting the chemistry of the drug and the lipids to the resulting thermodynamic properties remains of immediate importance. Here we report molecular dynamics (MD) simulation trajectories using the coarse-grained (CG) Martini force field. A wide, representative coverage of chemistry is provided: across solutes—exhaustively enumerating all 105 CG dimers—and across six phospholipids. For each combination, umbrella-sampling simulations provide detailed structural information of the solute at all depths from the bilayer midplane to bulk water, allowing a precise reconstruction of the potential of mean force. Overall, the present database contains trajectories from 15,120 MD simulations. This database may serve the further identification of structure-property relationships between compound chemistry and drug permeability.

## Abstract (summary)

**Table:** 

Measurement(s)	molecular dynamics trajectories
Technology Type(s)	computational modeling technique • molecular dynamics simulation
Factor Type(s)	Small organic molecules • phospholipid

Machine-accessible metadata file describing the reported data: 10.6084/m9.figshare.11733504

## Background & Summary

The passive permeation of small organic, drug-like molecules across phospholipid membranes has garnered much interest, not only to practically optimize pharmaceutical properties, but also as a more fundamental physical-chemistry problem^1^. The latter acts as a testbed to understand the molecular driving forces at play during a permeation process across a soft interface. A more robust understanding of the structure-property relationship can be obtained by screening across chemistries and systematically measuring the permeability coefficient from *in vitro* experiments^2,3^. Given the small size and apparent bias of databases of experimental compounds^4^, the perspective to harness computational methods at high throughput has been on the rise^5–9^.

**Fig. 1:**
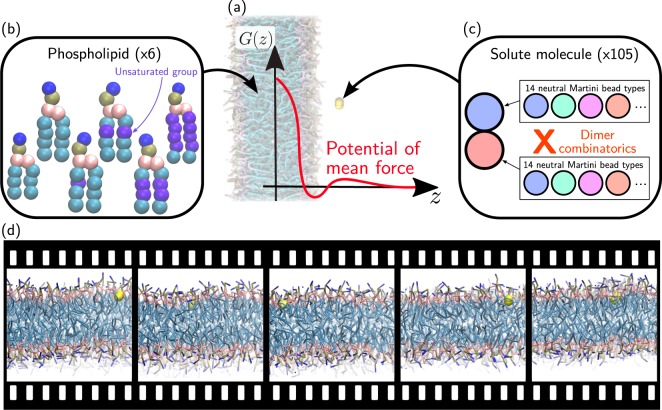
Drug-membrane computer simulation setup; screening over both phospholipids and solute molecules. (**a**) Background: Simulation setup of a solute (yellow) partitioning between water (not shown) and the lipid membrane. Foreground: Potential of mean force along the normal of the bilayer, *G*(*z*). (**b**) Lipid membrane: Cartoon representations of the five phospholipids, differing in the number of unsaturated groups. (**c**) Solute molecule: Combinatorics of all 105 CG Martini dimers. (**d**) The present dataset contains the trajectory of each MD simulation.

Permeation is described using the inhomogeneous solubility-diffusion model to yield a diffusion process in terms of a one-dimensional Smoluchowski equation along *z*—the normal to the membrane midplane. The resulting permeability coefficient, *P*, takes the form

1 $${P}^{-1}=\int dz\frac{exp[\beta G(z)]}{D(z)},$$

where ${\beta }^{-1}={k}_{B}T$ is the inverse temperature, *G*(*z*) is the potential of mean force (PMF), and *D*(*z*) is the local diffusivity. As such, knowledge of *G*(*z*) and *D*(*z*) enables an *in silico* estimation of *P*, which may be obtained from molecular dynamics (MD) simulations. While the direct estimation of these quantities from brute-force MD typically fails, enhanced-sampling methods have provided a robust strategy to estimate both *G*(*z*) and *D*(*z*). Equation 1 depends exponentially on *G*(*z*), but only linearly on *D*(*z*), making the latter quantity less critical—it was also found to depend rather weakly on the chemistry of the drug^10^. A large number of enhanced-sampling studies have demonstrated the capability to not only converge the PMF, but also to provide permeability coefficients that exhibit high correlation with experimental measurements^10–16^. An illustrative example of the PMF is shown in Fig. 1a, together with a cartoon of a phospholipid membrane in the background.

The *in silico* route is predictive and generalizable in that it does not rely on adjustable parameters: the main input of an MD simulation is the force field, often parametrized on properties unrelated to interactions with a phospholipid membrane^17,18^. Critically, this limits the danger of overfitting observed in statistical models^19^. The main downside of using MD simulations is the computational investment: atomistic simulations with explicit solvent typically require 10^5^ CPU-hours for a small molecule in a single-component lipid membrane^10,13,14,20^, hindering the prospects of running them at high-throughput.

We have recently proposed the use of coarse-grained (CG) models to tackle this problem. Coarse-graining can enable a more efficient sampling of the conformational space by lumping together atoms into super-particles or beads^21,22^. In particular we relied on the CG Martini model^23–25^, which is specifically tailored to reproduce the partitioning behavior of compounds in different environments—thus making it particularly well suited for permeability calculations. The modularity of Martini means that it constructs molecules based on a small set of bead types, each one encoding different chemical properties—mainly hydrophobicity, hydrogen-bonding, and charge (see Table 1 for details). We reported the systematic calculation of PMFs using umbrella sampling for *all* CG compounds made of one and two neutral beads (hereafter denoted unimers and dimers)^26^. This amounted to 14 unimers and 14 × 15/2 = 105 dimers (Fig. 1c). The thermodynamic parametrization of Martini yields accurate PMFs, as compared to reference curves from atomistic simulations, as well as remarkably-accurate permeability coefficients, as compared to reference simulations and experiments^27^. Because of the transferable nature of Martini, the CG model significantly reduces the size of chemical space, such that these 119 computer simulations offer estimates for more than 500,000 small molecules^26,27^. We more recently extended our approach to linear trimers and tetramers, demonstrating that the screening range can be significantly increased^28^. As such, Martini offers a robust methodology to run high-throughput computer simulations of drug-membrane permeability.

**Table 1:** Characteristics of non-charged Martini bead types.

Polarity	Type	HB	Δ*G*_Ol→W_
hydrophilic	P5	—	−2.1
	P4	—	−2.2
	P3	—	−2.1
	P2	—	−0.9
	P1	—	−0.5
neutral	Nda	d, a	0.6
	Nd	d	0.6
	Na	a	0.6
	N0	—	1.0
hydrophobic	C5	—	1.7
	C4	—	2.4
	C3	—	3.0
	C2	—	3.3
	C1	—	3.4

The table contains information about the bead-type name (Type), hydrogen-bonding capability (HB, “d” and “a” for donor and acceptor, respectively), and the octanol/water partitioning free energy (Δ*G*_Ol→W_ = Δ*G*_water_ − Δ*G*_octanol_, in units of kcal/mol), as reported elsewhere^38^.

The present database reports the full umbrella-sampling MD trajectories necessary to run PMF calculations for all Martini dimers in six different single-component phospholipid membranes. The 105 dimers inserted in 6 membranes amounts to 630 drug-membrane combinations (Fig. 1). Given that each PMF calculation relied on 24 umbrella sampling simulations, the present database contains 15,120 MD trajectories. The diversity of compound and lipid chemistries can offer unprecedented insight into the underlying thermodynamics^26,29^. Below we present an example use of the present database by displaying the tilt angle of each compound across membrane-insertion depth and compound chemistry. We believe that the raw MD trajectories provided for this breadth of chemistries will provide further insight into the structure-property relationships governing drug-membrane permeability.

## Methods

We follow previously established simulation protocols that are described in detail elsewhere^26^. In brief, we built symmetric, single-lipid bilayer membranes that contain 64 lipids per leaflet using the Insane script^30^. Table 2 informs on the composition of the various membrane systems that differ in the number of water beads. As it is common practice, we replaced at least 10% of the non-polarizable Martini waters by anti-freeze beads. Simulations were performed in Gromacs 4.6.6^31^ using the Martini force field with standard input parameters^32^. We ran simulations in the *NPT* ensemble at 300 K and 1 bar controlled by means of a stochastic velocity-rescaling thermostat^33^ and a Parrinello-Rahman barostat^34^, respectively. We performed umbrella sampling along the bilayer normal (*z*-axis) in a range from 0.0 to 4.1 nm at a step size of 0.1 nm by generating 24 windows in which the solute is centered via a harmonic biasing potential (*k* = 240 kcal/mol/nm^2^). For computational efficiency, each simulation box contained two solute compounds placed in different membrane leaflets. Each window included a sequence of minimization, heat-up, and equilibration runs prior to the production one, the latter being simulated for 1.2 · 10^5^ *τ* using a time step of *δt* = 0.02 *τ*, where *τ* (1 ps) refers to the model’s natural unit of time. PMF profiles were then reconstructed by means of the weighted histogram analysis method (WHAM)^35,36^, with error bars estimated from 100 bootstraps.

**Table 2:** Composition of single-lipid bilayer membranes.

Membrane	N_*L*_	N_*W*_	N_*AF*_
DAPC	128	2430	270
DIPC	128	2120	236
DLPC	128	1883	209
DOPC	128	1890	189
DPPC	128	2014	224
POPC	128	2014	224

N_*L*_ = number of lipid molecules, N_*W*_ = number of water beads, N_*AF*_ = number of anti-freeze beads.

DAPC = 1,2-diarachidonoyl-*sn*-glycero-3-phosphocholine.

DIPC = 1,2-dilinoleoyl-*sn*-glycero-3-phosphocholine.

DLPC = 1,2-dilauroyl-*sn*-glycero-3-phosphocholine.

DOPC = 1,2-dioleoyl-*sn*-glycero-3-phosphocholine.

DPPC = 1,2-dipalmitoyl-*sn*-glycero-3-phosphocholine.

POPC = 1-palmitoyl-2-oleoyl-*sn*-glycero-3-phosphocholine.

## Data Records

**Fig. 2:**
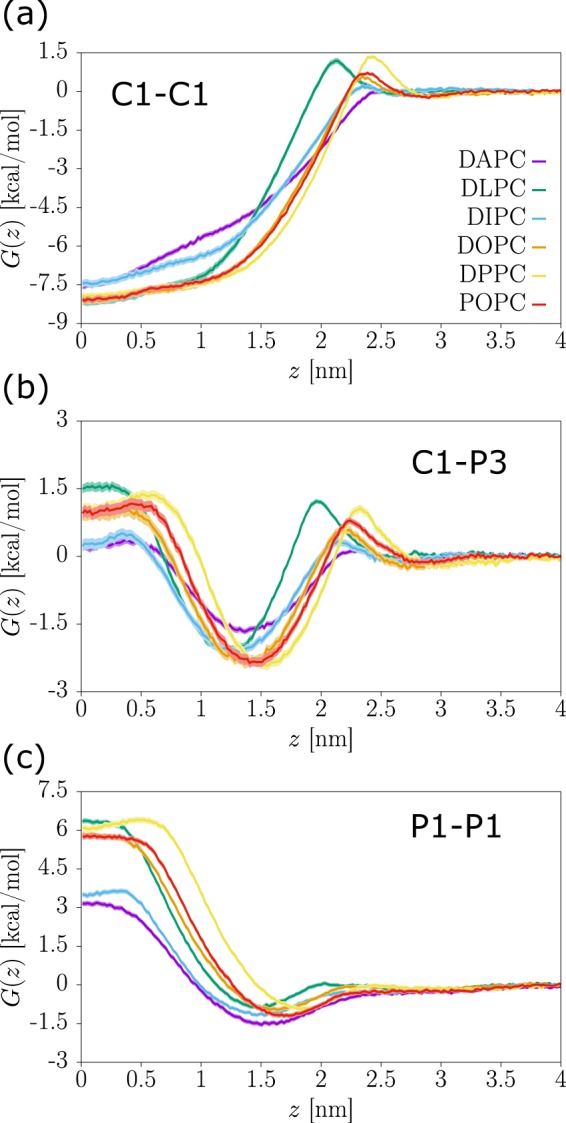
Examples of PMF profiles in different lipid environments. Three representative solutes are shown: (**a**) hydrophobic (C1-C1); (**b**) amphiphilic (C1-P3); and (**c**) polar (P1-P1). See labels for the different lipid types.

We provide datasets for MD trajectories of solute-membrane systems at a CG resolution for 105 solutes inserted in six different phospholipid bilayers^37^. Each dataset is denoted by the abbreviated name of the lipid and deposited as a single archive file, e.g., DPPC.tar.bz2. Within a dataset, there are 105 folders containing the trajectories and PMF profile of a particular solute, following the naming convention DIM_bead1-bead2, where bead1 and bead2 denote the relevant bead types following the standard Martini notation (see Table 1). For improved sampling we have systematically placed *two* solutes in each simulation box, always separated by a normal distance (i.e., only along *z*) of 4.1 nm. The trajectories obtained from umbrella sampling (US) are stored in sub-folders denoted us-x, where x takes values 0.0, 0.1, …, 2.4, corresponding to the reference depth in the bilayer of one of the two solutes. For instance, the folder us-2.4 contains two solutes restrained around *z*_1_ = 2.4 nm and *z*_2_ = −1.7 nm. The US sub-folders contain all necessary input files to repeat the production runs as well as the respective output files, including trajectories and observables. The sub-folder pmf contains the input files to perform WHAM reweighting in Gromacs and the output PMF profiles. Table 3 lists all files included in the sub-folders us-x and pmf together with a brief description of their purpose. In Fig. 2 we report typical PMF profiles obtained for hydrophobic (Fig. 2a), amphiphilic (Fig. 2b), and polar solute compounds (Fig. 2c) across all considered lipid environments.

**Table 3:** Supplied files and their purpose.

Folder/File	I/O	Description
us-x/		
equ.cpt	I	binary; checkpoint to extend a previous simulation
equ.gro	I	coordinates of the starting configuration
system.top	I	topology of the molecular system
martini_v2.2.itp	I	Force field and parameters
martini_v2.0_lipids.itp	I	Lipid force field
mol3.itp, mol3-2.itp	I	Solute force field
prod.mdp	I	simulation parameters
prod.tpr	I	binary; overall simulation information
prod.xtc	O	binary; trajectory
prod.edr	O	binary; various observables (e.g., energies)
prodx-umbrella0.xvg	O	time evolution of the solute CoMs along the *z* axis
prodf-umbrella0.xvg	O	time evolution of the pull forces along the *z* axis
pmf/		
tpr-files.dat	I	indexes paths to the tpr files
pullx-files.dat	I	indexes paths to the prodx files
bsResult.xvg	O	PMF profile with error bars

All files provided in text format unless specified otherwise. I/O = input/output. CoM = center of mass.

Our records indicate a computational investment to run heat-up, equilibration, and production simulations of roughly 0.5, 1, and 8 CPU-hours per umbrella, respectively. Summing up over all 15,120 MD simulations supplied, this amounts to roughly 150,000 CPU-hours to generate the present dataset.

## Technical Validation

A number of studies using the same simulation protocol have demonstrated the thermodynamic validity and accuracy of CG Martini simulations. Bereau and Kremer found a mean absolute error between experimental and Martini transfer free energies between water and octanol of 0.79 kcal/mol across 653 neutral small organic molecules—an excellent result given the minimalism of the model^38^. By further invoking relations between bulk transfer free energies, used as proxy for various environments of the membrane, we deduced a mean absolute error on features of our CG PMFs of approximately 1.4 kcal/mol^26^. This remarkable agreement had been earlier probed specifically for amino acids^39^. On a structural level, we showed that backmapping CG snapshots and running short atomistic MD simulations offered a significant speedup in convergence of the atomistic PMF calculations, suggesting that the conformational ensemble of the CG model adequately matches its atomistic counterpart^40^. Moreover, as the permeability coefficient depends exponentially on the PMF, reliable estimates of the latter prove necessary^13^. We showed that the accuracy of the PMFs obtained through Martini translated into excellent predictability for the permeability coefficient—roughly 1 log unit^27,29^.

## Usage Notes

**Fig. 3:**
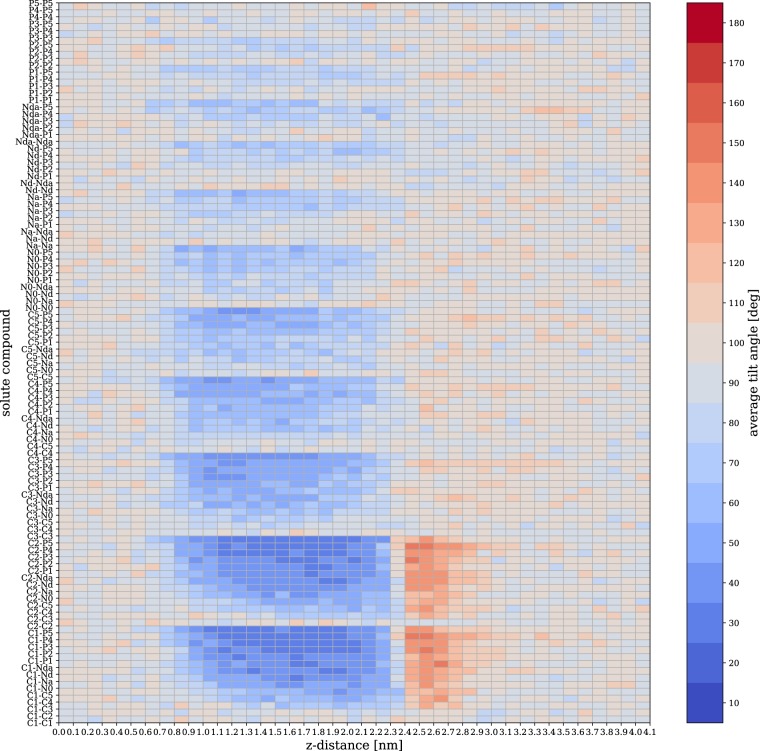
Average tilt angle in a DPPC bilayer as function of the *z*-distance across all 105 solute compounds.

The 15,120 MD trajectories in this dataset provide a rich amount of information. As an illustration, we focus on the orientation of the solute with respect to the normal of the membrane bilayer. We define a tilt angle *θ* between the bond vector of the solute and the normal vector of the membrane, oriented as to point from the the bilayer midplane to the membrane surface. Figure 3 displays the average tilt angle as a function of the depth *z* in a DPPC bilayer across all 105 solute compounds. The depicted angles are normalized to sin *θ* to account for the Jacobian of the transformation to spherical coordinates.

We find that solutes composed of two beads of identical or similar polarity display no preferred orientation, such that *θ* ≈ 90°. On the other hand, features appear for compounds that show a *difference* in polarity between the two beads of the solute. These features are markedly present in the range of depths 0.9 < *z* < 2.2 nm, which entails the lipid tail region. These amphiphilic solutes, such as C1-P5, show a strong preference for small tilt angles *θ* < 45°, where the more hydrophobic bead is facing the membrane core. The lack of features below *z* ≈ 0.9 nm is likely due to the force field’s interaction cut-off. In addition, strongly amphiphilic solutes also show orientational order at the membrane/water interface (2.4 < *z* < 2.7 nm), but with a flipped bond vector (*θ* ≈ 130°), i.e., the polar site now faces the membrane.

## Data Availability

No custom code was used to generate this dataset. Simulations were run using Gromacs 4.6.6, and all input files used to generate the trajectories are contained in the present dataset. The output data contains a number of binary files, all generated from Gromacs. Gromacs is available across many platforms and architectures (see Gromacs manual) and its output files can be read either from a compiled version of the freely-available code, or from other analysis modules, such as mdanalysis^41^ or mdtraj^42^. The WHAM-reconstructed PMFs were obtained by running a command that can be found in each DIM_bead1-bead2/pmf/bsResult.xvg file.
